# Bovine Milk Extracellular Vesicles (EVs) Modification Elicits Skeletal Muscle Growth in Rats

**DOI:** 10.3389/fphys.2019.00436

**Published:** 2019-04-16

**Authors:** Hailey A. Parry, C. Brooks Mobley, Petey W. Mumford, Matthew A. Romero, Cody T. Haun, Yufeng Zhang, Paul A. Roberson, Janos Zempleni, Arny A. Ferrando, Ivan J. Vechetti, John J. McCarthy, Kaelin C. Young, Michael D. Roberts, Andreas N. Kavazis

**Affiliations:** ^1^School of Kinesiology, Auburn University, Auburn, AL, United States; ^2^Department of Physiology, University of Kentucky College of Medicine, Lexington, KY, United States; ^3^Department of Nutrition and Health Sciences, University of Nebraska–Lincoln, Lincoln, NE, United States; ^4^Donald W. Reynolds Institute on Aging, University of Arkansas for Medical Sciences, Little Rock, AK, United States; ^5^Department of Cell Biology and Physiology, Edward Via College of Osteopathic Medicine-Auburn Campus, Auburn, AL, United States

**Keywords:** extracellular vesicles, RNA, muscle growth, mitochondria, oxidative stress

## Abstract

The current study investigated how bovine milk extracellular vesicles (EVs) affected rotarod performance and biomarkers of skeletal muscle physiology in young, growing rats. Twenty-eight-day Fisher 344 rats were provided an AIN-93G-based diet for 4 weeks that either remained unadulterated [EVs and RNA-sufficient (ERS; *n* = 12)] or was sonicated [EVs and RNA-depleted (ERD; *n* = 12)]. Prior to (PRE) and on the last day of the intervention (POST), animals were tested for maximal rotarod performance. Following the feeding period, the gastrocnemius muscle was analyzed at the histological, biochemical, and molecular levels and was also used to measure mitochondrial function and reactive oxygen species (ROS) emission. A main effect of time was observed for rotarod time (PRE > POST, *p* = 0.001). Terminal gastrocnemius mass was unaffected by diet, although gastrocnemius muscle fiber cross sectional area was 11% greater (*p* = 0.018) and total RNA (a surrogate of ribosome density) was 24% greater (*p* = 0.001) in ERD. Transcriptomic analysis of the gastrocnemius indicated that 22 mRNAs were significantly greater in ERS versus ERD (*p* < 0.01), whereas 55 mRNAs were greater in ERD versus ERS (*p* < 0.01). There were no differences in gastrocnemius citrate synthase activity or mitochondrial coupling (respiratory control ratio), although mitochondrial ROS production was lower in ERD gastrocnemius (*p* = 0.016), which may be explained by an increase in glutathione peroxidase protein levels (*p* = 0.020) in ERD gastrocnemius. Dietary EVs profiling confirmed that sonication in the ERD diet reduced EVs content by ∼60%. Our findings demonstrate that bovine milk EVs depletion through sonication elicits anabolic and transcriptomic effects in the gastrocnemius muscle of rapidly maturing rats. While this did not translate into a functional outcome between diets (i.e., rotarod performance), longer feeding periods may be needed to observe such functional effects.

## Introduction

Extracellular vesicles (EVs) are nanoparticle-sized lipid bilayer-coated vesicles that are secreted from all cells (Colombo et al., 2013, 2014; Yanez-Mo et al., 2015; Zempleni, 2017). EVs are known for carrying ‘cargo,’ such as ribonucleic acid (RNA), microRNA (miRNA), and transfer RNA (tRNA) which may be involved in regulating cellular signaling pathways (Mittelbrunn and Sanchez-Madrid, 2012; Baglio et al., 2015). The mechanism through which EVs release their cargo to act upon other targets is not completely understood, although it is accepted that the cargo of the EVs can interact locally and systemically (Valadi et al., 2007; Fry et al., 2017).

In 2012, a novel observation was made in which exogenous rice-borne miRNAs were present in serum and tissues of various animals which consumed a rice-based diet (Zhang et al., 2012). Zhang et al. (2012) reported rice miRNA 168a (MIR168a) is one of the most highly enriched exogenous plant miRNAs in the blood of Chinese subjects who consume diets rich in rice. Interestingly, MIR168a was shown to bind to the low-density lipoprotein receptor adapter protein 1 (ldlrap1) mRNA, inhibit ldlrap1 expression in the liver of mice, and thus decrease LDL levels in mouse plasma (Zhang et al., 2012). This was the first *in vivo* observation of physiological alterations occurring due to miRNAs obtained through the diet.

Researchers have also noted that orally obtained milk-EVs can withstand the digestive process and can be absorbed intact to affect downstream physiological processes (Zhou et al., 2012; Izumi et al., 2015; Pieters et al., 2015). Additionally, bovine milk-specific miRNA-143, -150, -378, 380-3p, and -1839 have been detected in human plasma following milk consumption (Shu et al., 2015; Manca et al., 2018). Our laboratory has previously reported that treatments with whey protein-derived EVs *in vitro* influences the appearance of bovine-specific miRNAs in C2C12 myotubes (Mobley et al., 2017c). Furthermore, these EVs increased skeletal muscle protein synthesis (MPS) and anabolism, potentially through gene regulation by the involvement of bovine-specific miRNA (Mobley et al., 2017c).

Endogenous miRNAs have previously been linked to muscle protein accretion (Zacharewicz et al., 2014) in addition to regulating mitochondrial function (Duarte et al., 2014). However, the ramifications of exogenous bovine milk-EVs delivery to peripheral tissues have been understudied. Therefore, the purpose of this study was to determine the potential role of bovine milk-derived EVs in affecting biomarkers of skeletal muscle physiology in maturing rats.

## Materials and Methods

### Animals and Experimental Procedures

All experimental procedures were approved by Auburn University’s Institutional Animal Care and Use Committee (IACUC, protocol #2017-3081). Twelve male and 12 female Fisher 344 rats at 28 days of age were purchased (Harlan Laboratories, Indianapolis, IN, United States) and allowed to acclimate in the animal housing facility for 1 week prior to experimentation. During acclimation, animals were provided standard rodent chow (SC; 24% protein, 58% CHO, 18% fat; Teklad Global #2018 Diet, Harlan Laboratories) and water *ad libitum* in a maintained ambient temperature and constant 12 h light: 12 h dark cycle. After acclimation, half the male (*n* = 6) and half the female rats (*n* = 6) were provided with a diet for 4 weeks that either remained unadulterated [EVs and RNA-sufficient (ERS; *n* = 12)] or was sonicated [EVs and RNA-depleted (ERD; *n* = 12)]. Both diets were based on the AIN-93G formula (Reeves et al., 1993) with modification previously reported by Zempleni’s laboratory (Baier et al., 2014; Leiferman et al., 2018). Importantly, only the milk ingredient of the ERD was ultrasonicated; all other ingredients were not altered.

The day prior to starting the dietary treatments and the last day of dietary treatments, rotarod performance was used to assess a combination of balance, grip strength, motor coordination, and muscular endurance using a single-lane device (ENV-571R; Med Associates, Inc., St. Albans, VT, United States) as previously described by our laboratory (Mobley et al., 2017b). All assessments took place during the beginning of the rat light cycle (0600–0800) whereby rats were placed on the device, and the motorized rotor was initiated at a progressive speed from 4.0 to 40.0 revolutions/min. An automated timer tracked time spent on the rod, and once the rats dismounted from the rod, a laser beam break stopped the timer.

The night prior to euthanasia, rats were food-deprived but provided water *ad libitum*. On the morning of necropsies (0500–0600) rats were transported from the campus vivarium to the School of Kinesiology and allowed to acclimate for 2–3 h. Animals were then injected with puromycin (5.44 mg/ml phosphate buffered saline; Ameresco, Solon, OH, United States) 30 min prior to euthanasia as previously detailed by our laboratory to assess MPS levels using the SUnSET method (Mobley et al., 2016, 2017b). Thereafter, rats were euthanized under CO_2_ gas in a 2 L induction chamber (VetEquip, Pleasanton, CA, United States). Following euthanasia, body mass was recorded, rats were rapidly dissected, and inguinal, mesenteric, and omental adipose tissue, liver, and hind limb skeletal muscle masses were recorded. The left and right gastrocnemius were used for the measurements described below.

### Histology

Approximately 30 mg of the gastrocnemius between the medial and lateral heads was embedded in cryomolds containing optimal cutting temperature (OCT) media (Tissue-Tek^®^, Sakura Finetek, Inc., Torrence, CA, United States). Cryomolds were frozen using liquid nitrogen-cooled isopentane and stored at -80°C. Methods for cryostat sectioning have been employed previously in our laboratory (Hyatt et al., 2015; Martin et al., 2016; Mobley et al., 2017a). Briefly, sections from OCT-preserved samples were cut at a thickness of 8 μm using a cryotome (Leica Biosystems, Buffalo Grove, IL, United States) and were adhered to positively-charged histology slides. Then, sections were air-dried, permeabilized, and blocked with Pierce Super Blocker (Thermo Fisher Scientific, Waltham, MA, United States). Sections were then incubated with a pre-diluted commercially available rabbit anti-dystrophin IgG antibody solution (GTX15277; Genetex, Inc., Irvine, CA, United States). Sections were then washed for and incubated in the dark with a secondary antibody solution containing Texas Red-conjugated anti-rabbit IgG (TI-1000; Vector Laboratories, Burlingame, CA, United States). Sections were washed, air-dried and mounted with fluorescent media containing 4,6-diamidino-2-phenylindole (DAPI; GTX16206; GeneTex, Irvine, CA, United States). Following mounting, slides were imaged using a 10× objective using a fluorescence microscope (Nikon Instruments, Melville, NY, United States). All images were captured by a laboratory technician who was blinded to diet groups. This staining method allowed the identification of cell membranes (detected by the Texas Red filter) and myonuclei (detected by the DAPI filter). Mean gastrocnemius fiber cross sectional area (fCSA) assessments were performed using custom-written pipelines in the open-sourced software CellProfiler^TM^ (Carpenter et al., 2006) whereby the number of pixels counted within the border of each muscle fiber was converted to a total area (μm^2^). Myonuclear counting was also performed using a custom pipeline in CellProfiler^TM^ as previously described by our laboratory (Mobley et al., 2017a; Haun et al., 2018). Approximately 350 fibers per animal were quantified for fCSA and myonuclear number.

### RNA Isolation and Transcriptomics

Approximately 30 mg of gastrocnemius between the medial and lateral heads was obtained and the tissue was homogenized in 1.7 mL microcentrifuge tubes containing 500 μL of Ribozol (Ameresco, Solon, OH, United States) via micropestle manipulation and RNA isolation was performed per manufacturer’s recommendations. Samples were then frozen at -80°C until RNA quantification. Total RNA per unit muscle weight was used as a surrogate for ribosome density as described by our laboratory and others (Nader et al., 2005; Martin et al., 2016; Mobley et al., 2016), and changes in total RNA were presumed to represent changes in ribosome density. A sub-fraction of isolated RNA (ERS *n* = 8, ERD *n* = 8; four males and four females per treatment) was shipped on dry ice for commercial mRNA and miRNA sequencing (LC Sciences, Houston, TX, United States).

Total RNA quality and quantity were assessed using Bioanalyzer 2100 and RNA 6000 Nano LabChip Kits (Agilent, Santa Clara, CA, United States), and all samples were confirmed to possess RNA Integrity Numbers > 7.0. Total RNA was subjected to poly(A) mRNA enrichment with poly-T oligo attached magnetic beads (Invitrogen, Carlsbad, CA, United States). Poly(A) mRNA fractions were then fragmented into small pieces using divalent cations under elevated temperature. The cleaved RNA fragments were subsequently reverse-transcribed to create the final cDNA library in accordance with strand-specific library preparation by dUTP method. The average insert size for the paired-end libraries was 300 ± 50 bp. Paired-end 2 bp × 150 bp sequencing was performed on an Illumina Hiseq 4000 platform following the vendor’s recommended protocol. Firstly, Cutadapt (Martin, 2011) and perl scripts were used to remove the reads that contained adaptor contamination, low quality bases and undetermined bases. Then sequence quality was verified using FastQC^1^. HISAT2 (Kim et al., 2015) was then used to map reads of the genome of *Rattus norvegicus* (Version v88). The mapped reads of each sample were assembled using StringTie (Pertea et al., 2015). Then, all transcriptomes from the 16 samples were merged to reconstruct a comprehensive transcriptome using perl scripts and gffcompare^2^. After the final transcriptome was generated, StringTie (Pertea et al., 2015) was used to measure expression levels for mRNAs by calculating FPKM values (FPKM = [total_exon_fragments/mapped_reads (millions) × exon_length (kB)]). Differentially expressed transcripts were considered meaningful between treatments if the following criteria were met: (a) average FPKM values for a given transcript from all 16 animals were > 1.0, (b) the fold-change score of a given mRNA was ± 1.5 fold between treatments, and (c) the un-adjusted *p*-value of a given mRNA between treatments was *p* < 0.01. Thereafter, the list of differentially expressed mRNA were entered into DAVID Bioinformatics (v 6.8) (Huang da et al., 2009a,b) to determine significantly annotated gene pathway differences that existed between treatments, and significance for this analysis was set at *p* < 0.05. It should be noted that a total of two mismatches were allowed for the mRNA sequencing data.

Small RNA libraries were generated from total RNA using the Illumina Truseq^TM^ Small RNA Preparation kit according to Illumina’s TruSeq^TM^ Small RNA Sample Preparation Guide1. The purified cDNA library was sequenced on the HiSeq 2500 platform in the 50 bp SE configuration following vendor’s instructions. Raw sequencing reads were obtained using Illumina’s Sequencing Control Studio software version 2.8 (SCS v2.8) following real-time sequencing image analysis and base-calling by Illumina’s Real-Time Analysis version 1.8.70 (RTA v1.8.70). The extracted sequencing reads were then used in the standard data analysis, which was performed using a proprietary pipeline script (ACGT101-miR v4.2; LC Sciences). Briefly, after the raw sequence reads, or sequenced sequences (sequ seqs) were extracted, a series of digital filters were employed to remove various un-mappable sequencing reads. In this step, the “impurity” sequences due to sample preparation, sequencing chemistry and processes, and the optical digital resolution of the sequencer detector were removed. Those remaining sequ seqs (filtered sequ seqs with lengths between 15 and 32 bases) were grouped by families (unique seqs) and were used to map with reference database files which contained pre-miRNA and mature miRNA sequences listed in the latest release of miRBase (version 22) or rat and bovine genomes based on the publically-available data. Given that only dozens of miRNAs were obtained following sequencing, differentially expressed miRNAs were considered meaningful between treatments if the un-adjusted *p*-value between treatments was *p* < 0.05. It should be noted that a total of two mismatches were allowed for the mRNA sequencing data.

### 20S Proteasome Activity Assay

Approximately 30 mg of gastrocnemius muscle between the medial and lateral heads was placed in 500 μL of ice-cold cell lysis buffer [Cell Signaling; 20 mM Tris-HCl (pH 7.5), 150 mM NaCl, 1 mM Na-EDTA, 1 mM EGTA, 1% Triton, 20 mM sodium pyrophosphate, 25 mM sodium fluoride, 1 mM β-glycerophosphate, 1 mM Na_3_VO_4_, 1 μg/mL leupeptin] and samples were then homogenized via micropestle manipulation, and insoluble proteins from homogenates were removed with centrifugation at 500 *g* for 5 min. Protein determination on cell lysis homogenates was subsequently performed with a BCA Protein Assay Kit (Thermo Fisher Scientific). Homogenates were then stored at -80°C. Forty μg of protein were assayed for 20S proteasome activity as previously described using commercially available fluorometric kits (APT280; Millipore Sigma, Burlington, MA, United States) per the manufacturer’s instructions and previously described by our laboratory (Mobley et al., 2017b).

### Western Blotting

Homogenates were prepared for Western blotting at 1 μg/μL. Subsequently, 15 μL of prepped sample were separated by SDS-polyacrylamide gel electrophoresis. After electrophoresis, proteins were transferred to polyvinylidene difluoride membranes (Bio-Rad Laboratories, Hercules, CA, United States). Membranes were Ponceau stained and imaged for signal normalization using a gel documentation system and associated densitometry software (UVP, Upland, CA, United States). Membranes were then blocked and then incubated with a primary antibody directed against the protein of interest [puromycin, clone 12D10, Millipore-Merck KGaA, Darmstadt, Germany and superoxide dismutase 1 (SOD1), GTX100554; superoxide dismutase 2 (SOD2), GTX116093; catalase (CAT) GTX110704; glutathione peroxidase (GPX), GTX116040; peroxisome proliferator-activated receptor gamma coactivator 1-alpha (PGC-1α) GTX37356; GeneTex and 4-hydroxynonenal-conjugated proteins (4HNE) ab46545, Abcam, Cambridge, MA, United States]. Membranes were then incubated with a secondary antibody and then imaged using an enhanced chemiluminescent reagent, and band densitometry was performed using a gel documentation system and associated densitometry software (UVP). In addition, protein carbonyls were determined using the Oxyblot kit (EMD Millipore, Billerica, MA, United States) as outlined by the manufacture instructions and previously reported by our laboratory (Kephart et al., 2017). Briefly, gastrocnemius homogenates were derivatized to 2,4-dinitrophenylhydrazone by a reaction with 2,4-dinitrophenylhydrazine. The DNP-derivatized protein samples were separated by polyacrylamide gel electrophoresis, transferred to polyvinylidene difluoride membranes, and incubated with the primary antibody provided in the kit. Following incubation with primary antibodies, membranes were then incubated with secondary antibodies. All membranes were then developed using an enhanced chemiluminescent reagent, and band densitometry was performed as described above.

### Mitochondrial Isolation, Respiration, and ROS Production

Differential centrifugation was used to isolate gastrocnemius mitochondria as previously described by our laboratory (Kavazis et al., 2010). Mitochondrial oxygen consumption was measured in a respiration chamber maintained at 37°C (Hansatech Instruments, Norfolk, United Kingdom). Isolated mitochondria were incubated with 1 mL of respiration buffer containing 100 mM KCl, 5 mM KH_2_PO_4_, 1 mM EGTA, 50 mM MOPS, 10 mM MgCl_2_, and 0.2% BSA at 37°C in a water-jacketed respiratory chamber with continuous stirring. Flux through complex I was measured using 2 mM pyruvate, 2 mM malate, and 10 mM glutamate. The maximal respiration (state 3) was defined as the rate of respiration in the presence of ADP, initiated by adding 5.0 μL of a 50 mM solution of ADP in the respiration chamber to raise the known concentration of ADP to 0.25 mM. State 4 respiration was recorded following the phosphorylation of ADP. The respiratory control ratio (RCR) was calculated by dividing state 3 by state 4 respiration.

Mitochondrial reactive oxygen species (ROS) production was determined using Amplex red (Molecular Probes, Eugene, OR, United States). The assay was performed at 37°C in 96-well plates with succinate as the substrate. Specifically, this assay was developed on the concept that horseradish peroxidase catalyzes the H_2_O_2_-dependent oxidation of non-fluorescent Amplex red to fluorescent resorufin red, and it is used to measure H_2_O_2_ as an indicator of superoxide production. SOD was added at 40 U/mL to convert all superoxide to H_2_O_2_. Using a multiwell-plate reader fluorometer (BioTek Synergy H1 Multi-Mode Reader; BioTek), we monitored resorufin formation at an excitation wavelength of 545 nm and a production wavelength of 590 nm. The level of resorufin formation was recorded every 5 min for 15 min, and H_2_O_2_ production was calculated with a standard curve.

### Citrate Synthase Activity Assay

Gastrocnemius homogenate citrate synthase activity was measured as a function of the increase in absorbance from 5,5′-dithiobis-2-nitrobenzoic acid reduction (Trounce et al., 1996). Enzyme activities were normalized to total protein levels.

### Dietary EVs Authentication

To validate the diets, the following analyses were performed. ERS and ERD samples were pulverized into a powder using a standard mortar and pestle. Approximately, 40 g of powder from each diet were used for analysis. Based on a protocol by Yamada et al. (2012), each sample was subjected to centrifugation at 5,000 *g* for 30 min at 4°C in order to remove any milk fat globules and any other solid debris. Following centrifugation, approximately 20 mL of the aqueous supernatant was removed and filtered through a sterile 0.2 μm polyesthertone membrane filter (VWR International, Radnor, PA, United States). Using a membrane-based affinity, EVs isolation kit (exoEasy Maxi Kit, Qiagen, Hilden, Germany), EVs were isolated from the supernatants and processed according to manufacturer’s instructions. Isolated EVs samples were analyzed at 11 different positions for size and concentration using nanoparticle tracking analysis (NTA) with the ZetaView PMX 110 (Particle Metrix, Meerbusch, Germany) and quantified using the corresponding software (ZetaView 8.02.28) (Mehdiani et al., 2015).

Following visual analysis, samples were measured and analyzed for RNA and protein content. EVs samples were lysed with Trizol LS Reagent (Thermo Scientific) and incubated at room temperature for 5 min. Then, 1-bromo-3-chloropropane (Millipore Sigma) was added to each sample and subjected to centrifugation at 12,000 *g* for 15 min at 4°C. The aqueous/upper phase of the lysates was retained and processed according to manufacturer’s instructions utilizing a standard exosomal RNA isolation kit (exoRNeasy Serum/Plasma Midi Kit, Qiagen) and RNA concentrations for each sample were quantified using a nanodrop (NanoDrop 2000, Thermo Fisher). Following RNA quantification, small RNA and miRNA content was assessed using a Small RNA Analysis Kit (Agilent), analyzed and quantified using the corresponding bioanalyzer machine and software (Bioanalyzer 2100, Agilent).

For protein isolation, the interphase and lower/Trizol phase from each sample was retained and processed per manufacturer instructions. Exosomal protein concentrations were prepared at 4 μg/μL for Western blotting. Briefly, samples were separated by SDS-polyacrylamide gel electrophoresis and then transferred onto nitrocellulose membranes (BioRad). Membranes were subsequently probed for heat shock protein 70 (HSP70) and cluster of differentiation (CD81) (EXOAB-Hsp70A-1; EXOAB-CD81A-1; System Biosciences, Palo Alto, CA, United States). Membranes were then incubated with a secondary antibody and then imaged using an Odyssey^®^ Infrared Imaging system (LI-COR Biotechnology, Lincoln, NE, United States). Protein profiling was assessed using Coomassie Brilliant Blue (BioRad) to evaluate whether EVs isolated from each diet displayed similar or different protein compositions.

### Statistical Analysis

All data in figures and tables are presented as means ± standard deviation. Figures also contain individual data points for each animal. Except for transcriptomic and miRNA data (statistical analyses discussed above), independent samples *t*-tests were used to determine if between-treatment differences existed. Additionally, rotarod performance time was compared between treatments over time using a 2 × 2 repeated measures analysis of variance, and a least significant difference *post hoc* was adopted *a priori* if the interaction was significant. Statistical significance for all hypothesis testing was set at *p* < 0.05.

## Results

### Body and Organ Masses and Food Consumed

Body and organ masses as well as food consumed are presented in Table 1. Terminal body mass, adipose tissues, liver, and hind limb skeletal muscle masses did not differ between treatments (*p* > 0.05). The only variable that significantly differed between treatments was food consumed (ERS > ERD, *p* = 0.026).

**Table 1:** Body and organ masses and food consumption and efficiency.

Variable	ERS	ERD	*p*-value
	(*n* = 12)	(*n* = 12)	
Terminal body mass (g)	162 ± 30	162 ± 25	0.979
Inguinal adipose tissue (g)	1.53 ± 0.59	1.72 ± 0.57	0.417
Mesenteric adipose tissue (g)	1.48 ± 0.57	1.57 ± 0.28	0.618
Omental adipose tissue (g)	0.38 ± 0.15	0.39 ± 0.10	0.865
Liver (g)	6.48 ± 1.73	6.39 ± 1.20	0.898
Right leg gastrocnemius (g)	0.78 ± 0.12	0.77 ± 0.10	0.681
Right leg plantaris (g)	0.15 ± 0.02	0.15 ± 0.02	0.904
Right leg soleus (g)	0.062 ± 0.011	0.062 ± 0.006	0.730
Total food consumed (g)	232 ± 17	203 ± 38	**0.026**
Mass gained over 28-days period (g)	94.6 ± 27.7	96.3 ± 25.1	0.873
Feed efficiency (g mass gained/g food consumed)	0.41 ± 0.13	0.47 ± 0.07	0.168

*ERS, exosome and RNA-sufficient; ERD, exosome and RNA-depleted. Bold value indicates *p* < 0.05*.

### Rotarod Performance

**Figure 1:**
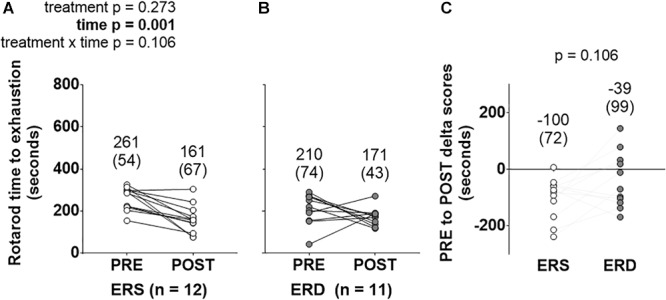
Rotarod performance between treatments over time. These data show rotarod performance prior to (PRE) and following (POST) the 28-day feeding intervention in ERS **(A)** and ERD **(B)**. **(C)** Shows PRE to POST delta scores. Notably, only a significant main effect of time was observed. Group mean ± parenthesized standard deviation values are presented above all data points. ERS, EVs and RNA-sufficient; ERD, EVs and RNA-depleted.

PRE- and POST-intervention rotarod performance is presented in Figure 1. There was a main effect of time (POST > PRE, *p* = 0.001), but no significant treatment or treatment × time interaction.

### Markers of Anabolism

**Figure 2:**
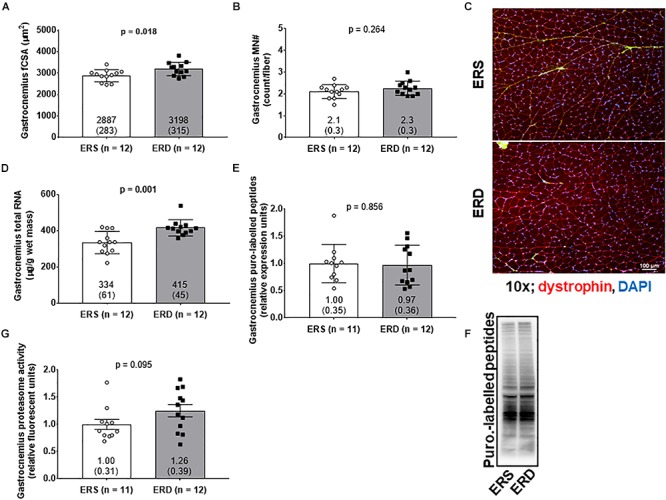
Markers of anabolism between treatments. These data show gastrocnemius muscle fiber cross-sectional area (fCSA) **(A)**, myonuclear number (MN#) **(B)**, total RNA indicative of ribosomal density **(D)**, puromycin-labeled peptides indicative of relative muscle protein synthesis (MPS) levels **(E)**, and proteasome activity **(G)**. **(C)** contains a 10x representative objective from each group, and **(F)** contains a representative puromycin Western blot. Group mean ± parenthesized standard deviation values are presented, and individual values are also presented. ERS, EVs and RNA-sufficient; ERD, EVs and RNA-depleted.

Gastrocnemius fCSA values were greater in ERD versus ERS (*p* = 0.018; Figure 2A,C), and total RNA levels were greater in ERD versus ERS (*p* = 0.001; Figure 2D). There were no significant differences in gastrocnemius myonuclear number (Figure 2B), puromycin-labeled peptides indicative of relative MPS levels (Figure 2E,F), or proteasome activity (Figure 2G).

### Transcriptome and miRNA

**Figure 3:**
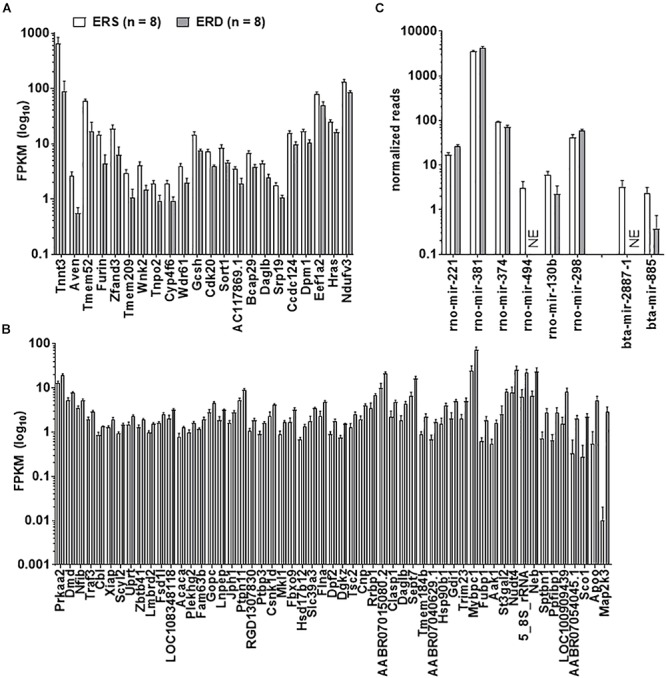
Transcriptome and miRNA differences between treatments. Data in the figure demonstrate transcriptome-wide and miRNA differences between treatments. Only 22 mRNAs were greater in ERS versus ERD rats (thresholds for significance listed in Section “Materials and Methods”) **(A)**, whereas 55 mRNAs were greater in ERD versus ERS rats **(B)**. **(C)** Demonstrates that six rat miRNAs were different between treatments, whereas two bovine miRNAs were different. ERS, EVs and RNA-sufficient; ERD, EVs and RNA-depleted; NE, not expressed at a detectable level.

Twenty-two gastrocnemius mRNAs were significantly greater in ERS versus ERD (Figure 3A), whereas 55 mRNAs were significantly greater in ERD versus ERS (Figure 3B). Bioinformatics analysis using DAVID (v6.8) indicated that no annotated gene pathways were significantly greater in ERS versus ERD. However, the following annotated gene pathways were greater in ERD versus ERS: (a) cadherin binding involved in cell–cell adhesion (5 mRNAs, *p* = 0.002), (b) actinin-type, actin-binding, conserved site (3 mRNAs, *p* = 0.001), (c) fatty acid biosynthetic process (3 mRNAs, *p* = 0.005), (d) protein kinase activity (5 mRNAs, *p* = 0.006), and (e) binding site:ATP (4 mRNAs, *p* = 0.048). Only six rat gastrocnemius miRNAs were significantly different between ERS and ERD (Figure 3C), while only two bovine miRNAs were different between treatments (ERS > ERD for bta-mir-2887-1 and bta-mir-885, *p* < 0.05; Figure 3C).

### Citrate Synthase Activity, Mitochondrial Respiration, and Mitochondrial ROS Production

**Figure 4:**
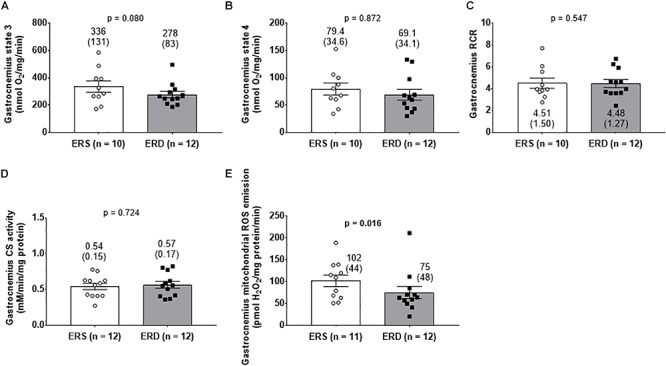
Mitochondrial markers between treatments. Gastrocnemius state 3 respiration **(A)**, state 4 respiration **(B)**, and respiratory control ratio (RCR) **(C)**. Citrate synthase (CS) activity reflective of mitochondrial volume **(D)**, and mitochondrial reactive oxygen species (ROS) emission **(E)**. Group mean ± parenthesized standard deviation values are presented, and individual values are also presented. ERS, EVs and RNA-sufficient; ERD, EVs and RNA-depleted.

There were no differences in gastrocnemius state 3 (Figure 4A), state 4 (Figure 4B), RCR (Figure 4C), or citrate synthase activity (Figure 4D). Interestingly, gastrocnemius mitochondrial ROS emission was greater in ERS versus ERD (*p* = 0.016; Figure 4E).

### Antioxidant Levels and Oxidative Damage Markers

**Figure 5:**
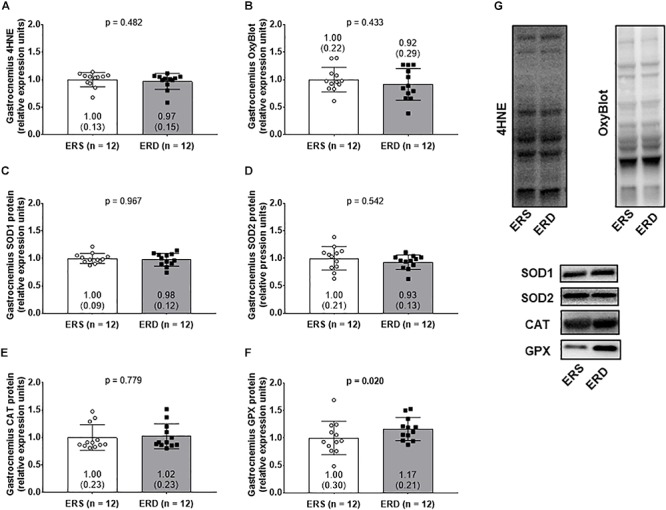
Antioxidant and oxidative damage markers between treatments. Gastrocnemius 4-hydroxynonenal (4HNE) levels **(A)**, protein carbonyl levels determined via the OxyBlot method **(B)**, superoxide dismutase 1 (SOD1) protein levels **(C)**, superoxide dismutase 2 (SOD2) protein levels **(D)**, catalase (CAT) protein levels **(E)**, and glutathione peroxidase (GPX) protein levels **(F)**. **(G)** Contains representative Western blots for each target. Group mean ± parenthesized standard deviation values are presented, and individual values are also presented. ERS, EVs and RNA-sufficient; ERD, EVs and RNA-depleted.

No significant differences existed for gastrocnemius 4HNE levels (Figure 5A), protein carbonyl levels (Figure 5B), SOD1 protein levels (Figure 5C), SOD2 protein levels (Figure 5D), or CAT protein levels (Figure 5E). Interestingly, GPX protein levels were greater in gastrocnemius ERD versus ERS (*p* = 0.020) (Figure 5F). Representative Western blots for each target are shown in Figure 5G.

### Dietary EVs Profile

**Figure 6:**
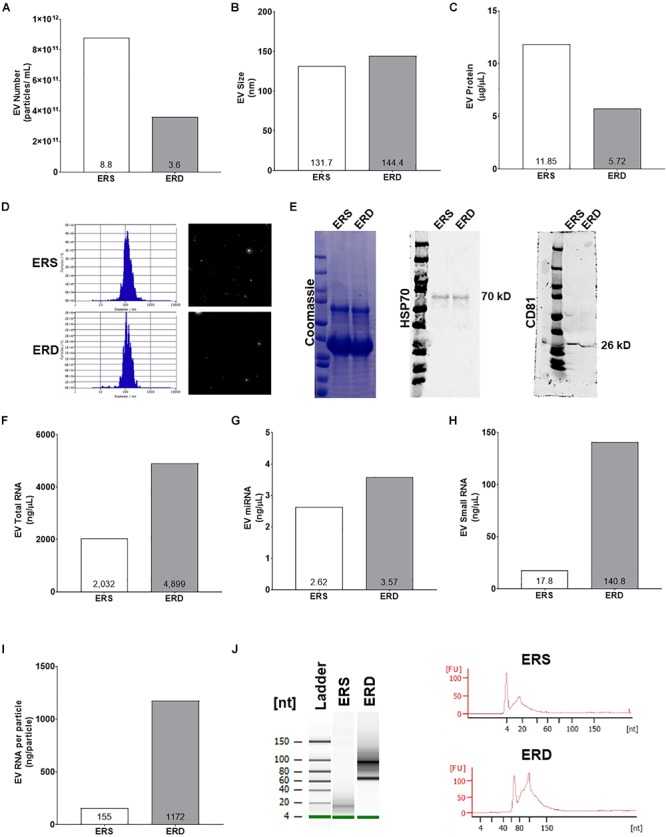
Extracellular vesicles authentication and cargo analysis between the ERS and ERD diets. These data show diet based bovine milk-derived EVs number **(A)**, EVs size **(B)**, EVs protein content **(C)**, ZetaView particle histograms and representative images **(D)**, representative Western blots for Coomassie, heat shock protein 70 (HSP70), and cluster of differentiation 81 (CD81) **(E)**, EVs total RNA content **(F)**, EVs miRNA content **(G)**, EVs small RNA content **(H)**, EVs RNA content per particle number **(I)**. **(J)** Contains representative gel and electropherograms from Bioanalyzer analysis. ERS, EVs and RNA-sufficient; ERD, EVs and RNA-depleted.

Extracellular vesicles profiling using the NTA via ZetaView capabilities indicated that ERD diet had less particles per mL (Figure 6A) and similar particle size (Figure 6B,D) compared to ERS diet. Additionally, EVs protein was lower in the ERD diet (Figure 6C). The protein profile of the EVs in each respective diet as assessed by Coomassie staining was similar and the presence of HSP70 and CD81 further confirmed the presence of EVs in both diets (Figure 6E). EVs total RNA was different between the diets (Figure 6F) and further RNA analyses revealed that miRNA and small RNA content was notably greater in the ERD compared to ERS diet (Figure 6G,H). The amount of RNA per EVs was ∼7.5-fold greater in the ERD versus ERS diet (Figure 6I). Bioanalyzer representative gel and electropherograms (Figure 6J) illustrate that there is a considerable shift in the small RNA profile with sonication.

## Discussion

While dietary milk EVs have been shown to be less enriched in skeletal muscle following oral gavage compared to other tissues (Leiferman et al., 2018; Manca et al., 2018), we have previously demonstrated that whey protein-derived EVs elicit an anabolic effect on C2C12 myotubes *in vitro* (Mobley et al., 2017c). Thus, the aim of this study was to comprehensively examine how bovine milk EVs modification through sonication affected rotarod performance and numerous biomarkers of skeletal muscle physiology *in vivo*.

One of the most intriguing findings of this study is that rats fed the ERD diet exhibited features of gastrocnemius muscle anabolism (i.e., increased fCSA and increased ribosome density). It is also interesting that more gastrocnemius transcripts were up-regulated in gastrocnemius muscles from ERD versus ERS rats, and bioinformatics showed processes related to cadherin binding, fatty acid biosynthesis, and protein kinase activity were upregulated in ERD-fed rats. Nonetheless, it is unlikely the gastrocnemius transcriptome signature in ERD-fed rats was due to bovine miRNA differences between diets given that only 2 bovine miRNAs were differentially expressed in the gastrocnemius between treatments (ERS > ERD for bta-mir-2887-1 and bta-mir-885). Importantly, and contrary to our hypothesis that the ERD diet would elicit negative effects on markers in anabolism in growing rats, we demonstrate that the aforementioned markers of anabolism were enhanced in ERD-fed rats. Indeed, these findings are difficult to explain given that only one other study has examined the effects of ERD feeding on growing mice (Leiferman et al., 2018). Notably, these authors fed 21-day old C57BL/6 male and female mice ERS or ERD diets for 6 weeks and reported minimal differences in quadriceps muscle mRNA transcripts between dietary treatments (e.g., Tmem100 mRNA levels were greater in ERD rats, while Socs2 mRNA levels were down-regulated in this group). The total RNA/mg muscle values (i.e., ribosome density) being significantly greater in ERD-fed rats in this study is in partial agreement with the aforementioned study by Leiferman et al. (2018) in that their transcriptomic-bioinformatics results suggest that skeletal muscle transcripts related to ribosome biogenesis are enriched in mice fed the ERD diet. However, it is curious that our transcriptome-wide comparisons between our rats and the mice in that study demonstrated no commonly affected mRNAs between diets. One clear difference between studies was that the paper by Leiferman et al. (2018) focused on transcriptome-wide effects of ERS versus ERD feeding in mice, whereas the current study thoroughly examined histological (i.e., fCSA), biochemical (i.e., MPS levels), and molecular alterations (i.e., proteasome activity and ribosome density) related to skeletal muscle anabolism. Thus, we contend that this is the first study to comprehensively phenotype the skeletal muscle effects of ERD-feeding *in vivo*.

The observed anabolic effects of ERD feeding could be due to one of the following: (a) the depletion of dietary EVs in the ERD diet via sonication leads to a de-repression signal independent of bovine miRNAs which facilitates muscle fiber growth, or (b) while sonication reduced EVs number, there was an appreciable RNA enrichment of the remaining EVs; thus, the modulation of dietary EVs through sonication may have further promoted dietary EVs-tissue signaling via exosomal RNA content (not miRNA content) which promoted muscle fiber growth. While no direct mechanism of action related to either phenomena was elucidated, these current results provide preliminary evidence that modulating bovine milk EVs via sonication may alter skeletal muscle physiology in rapidly maturing rats and warrant future research in other rodent and human models. Also, it is important to note that sonication of the milk to produce the ERD diet and subsequent consumption of this diet might have altered the microbiota of the ERD animals; however, this was not investigated in the current study. Notwithstanding, recent evidence has demonstrated that ERD-fed mice display an altered gut microbiome (Zhou et al., 2018). Thus, future research could potentially examine if milk-derived EVs affect different constituents of the gut microbiome, and whether this could have secondary effects on skeletal muscle mass regulation.

Regarding gastrocnemius mitochondrial adaptations to each diet, the following was observed: (a) mitochondrial volume assessed via citrate synthase activity was not different between dietary treatments, (b) mitochondrial ROS production significantly decreased in the gastrocnemius in ERD-fed rats with a concurrent increase in antioxidant GPX protein content, and (c) mitochondrial function (determined by RCR) was not different between dietary treatments. The lack of between-treatment differences in citrate synthase activity is consistent with the PGC-1α observations (data not shown), in which no change in protein expression was detected in the mitochondrial biogenesis marker between groups. Interestingly, gastrocnemius mitochondrial state 3 respiration with the complex I substrates decreased, albeit not significantly (*p* = 0.080), in the ERD rats. Additionally, we report mitochondrial ROS emission decreased in the gastrocnemius of ERD rats (*p* = 0.016). In this regard, mitochondrial state 3 respiration is the functional measure of maximal ATP production, and complex I is one of the sites which ROS emission occurs (Quinlan et al., 2013). Elevations in the NADH/NAD^+^ ratio or reverse electron transport are two proposed mechanisms which may increase complex I ROS production (Murphy, 2009). Therefore, the decrease in ROS emission may be explained in part by the decrease in maximal ATP production at complex I. It is possible that this decrease may be due to changes in the proposed mechanisms of ROS emission, however, more research is needed to elucidate this process (Goncalves et al., 2015). Furthermore, when the free radical superoxide is produced, it is rapidly converted to hydrogen peroxide and then GPX converts hydrogen peroxide to water (Pandey and Rizvi, 2010). Hence, the GPX protein elevations observed in ERD-fed rats may also contribute to the decrease in mitochondrial ROS emission. While the current evidence is preliminary, these results suggest that growing rats that consumed the ERD diet had lower mitochondrial ROS emission and higher endogenous antioxidant protein levels (i.e., GPX), but no significant changes in the two oxidative damage markers measured. The mechanism through which the ERD diet affects skeletal muscle oxidative stress markers remains to be determined and warrants future research. In addition, the only significant difference observed between male and female rats was a difference for state 3 and state 4 mitochondrial respiration (data not shown). Specifically, female rats had significantly lower state 3 and state 4 mitochondrial respiration rates compared to male rats independent of diet.

Our study possesses inherent limitations. First, only young and rapidly maturing rats were examined, and the feeding intervention was relatively short. As a result, our observations may not carry over into adult rodents and/or humans. Second, given the lack of data on how bovine milk EVs affects physiology *in vivo*, this study was exploratory in nature. For this reason, resources were devoted to exploring as many muscle biomarkers as possible. However, in-depth follow-up analyses were not performed (e.g., determining which molecule in ERD facilitated adaptations). It remains to be determined how the ERD diet mechanistically facilitated increases in muscle fCSA and ribosome content as well as delineating the mechanism by which skeletal muscle GPX and ROS levels were altered in ERD-fed rats. Notwithstanding, these preliminary data will certainly guide future mechanistic research efforts and provide proof of concept support that modulating bovine EVs may impact skeletal muscle physiology. It is also notable that the ERD diet, while depleted in EVs number, presented an enrichment in exosomal RNA levels. To study the effects of EVs depletion along with EVs RNA depletion, other methods beyond sonication may be needed (e.g., microwaving methods or sonication with detergent, etc.). It is also noteworthy mentioning that there are there are no established criteria that definitely allow for the distinction between EVs and small microvesicles (Nieuwland et al., 2018). Hence, the ERD food may be replete in both EVs and non-exosomal EVs. Finally, one interesting observation was that gastrocnemius fCSA increases in ERD-fed rats was not accompanied by proportional gastrocnemius weight increases. While this observation is difficult to explain, it is notable that other researchers in the field have posited that macro observations of hypertrophy, at times, poorly align with microscopic observations (Gordon, 1967). Herein, such findings may be due to the ERD feedings altering phenomena such as muscle fiber splitting during development, or eliciting an increase in sarcoplasmic expansion (and thus fCSA) which would increase muscle fiber size while not drastically affecting muscle weight. In this regard, our discrepant findings in ERD rodents is interesting and can lead to future avenues related to these research questions.

## Conclusion

Sonication is a newer approach to understanding how EVs obtained through the diet may affect the physiology of an organism. Notably, the changes observed in gastrocnemius fCSA, transcriptome, miRNA, and oxidative stress markers in rats suggest that modulating bovine milk-derived EVs may affect skeletal muscle physiology.

## Ethics Statement

All experimental procedures were approved by Auburn University’s Institutional Animal Care and Use Committee (IACUC, protocol #2017-3081).

## Author Contributions

AK, MDR, KY, and JZ contributed conception and design of the study. HP, CM, PM, MAR, CH, YZ, PR, and IV contributed to data collection, writing, and editing. JZ, AF, and JM contributed to the investigation, methodology, and editing. HP, CM, MDR, and AK wrote the first draft of the manuscript. All authors contributed to manuscript revision, read and approved the submitted version.

## Conflict of Interest Statement

JZ serves as a consultant for PureTech Health, Inc. The remaining authors declare that the research was conducted in the absence of any commercial or financial relationships that could be construed as a potential conflict of interest.

## Footnotes

1 www.bioinformatics.babraham.ac.uk/projects/fastqc/

2 https://github.com/gpertea/gffcompare/
